# Mitigating side channel attacks on FPGA through deep learning and dynamic partial reconfiguration

**DOI:** 10.1038/s41598-025-98473-3

**Published:** 2025-04-21

**Authors:** Sesibhushana Rao Bommana, Sreehari Veeramachaneni, Syed Ershad, MB Srinivas

**Affiliations:** 1 Department of Electrical & Electronics Engineering, BITS Pilani Hyderabad Campus, Hyderabad, 500078 India; 2 Department of Information Technology, Sri Sivasubramaniya Nadar College of Engineering, Chennai, 600020 India; 3 Department of Electronics and Communication Engineering, Aditya University, Kakinada, 533437 India

**Keywords:** Deep learning, SCA, DPR, FPGA, Engineering, Mathematics and computing

## Abstract

This paper introduces a framework that combines Deep Learning (DL) models and Dynamic Partial Reconfiguration (DPR) in Field Programmable Gate Arrays (FPGA) to mitigate Side Channel Attacks (SCA). Traditional static defense mechanisms often fail to fully mitigate SCA because they lack the ability to adapt dynamically to attacks. The proposed approach overcomes this limitation by adaptively reconfiguring the FPGA resources in real-time, disrupting the SCA patterns, and reducing the effectiveness of potential attacks. One of the notable advantages of this approach is its ability to defend against side-channel attacks while the FPGA design is operational. The framework accomplishes this by reconfiguring the FPGA resources to optimize response times, achieving latency levels beyond the reach of traditional static defense mechanisms. In particular, this study concentrates on mitigating power side-channel attacks, highlighting the resilience of the DL-DPR integration. Beyond its demonstrated efficacy against power SCA, the proposed framework can be extended to be adaptable to other types of side-channel attacks, making it a potential solution for hardware security. The integration of DL models allows for sophisticated threat analysis, while DPR provides the flexibility to implement countermeasures dynamically. Experimental results show that the latency from detection to mitigation is within 20 clock cycles. This combination represents a paradigm shift in securing hardware systems, moving from reactive to proactive defense mechanisms. The framework’s real-time adaptability ensures it stays ahead of attackers, continuously evolving to neutralize new threats. The findings presented in this paper underscore the potential of combining Artificial Intelligence (AI) and FPGA technologies to redefine hardware security. By addressing detection and mitigation in a unified framework, the proposed methodology significantly enhances the resilience of FPGA designs and lays the groundwork for future research in adaptive security mechanisms.

## Introduction

Hardware security^1^ protects physical computing devices and the data they process from malicious attacks, unauthorized access, and tampering. Hardware security focuses on the physical components of computers, such as processors, memory, and circuits, and specialized hardware modules like Trusted Platform Modules (TPM) and Hardware Security Modules (HSM). The primary goal of hardware security is to create secure, tamper-resistant systems that can defend against digital attacks (e.g., hacking attempts) and physical attacks (e.g., physical tampering with chips). Techniques in this domain include cryptographic hardware design, side-channel attack mitigation, secure boot processes, and hardware encryption. Innovations like secure enclaves, which isolate sensitive data, and hardware-based encryption keys further reinforce these protections by keeping critical data and processes separate from other system functions.

Side-channel attacks (SCA)^2^ pose a significant security challenge by allowing attackers to exploit indirect information from a system to gain unauthorized access to sensitive data. Unlike conventional attacks that target system vulnerabilities directly, SCAs rely on analyzing unintended “leakages” from hardware components. These leakages include timing variations, power usage patterns, electromagnetic emissions, and acoustic signals. Such information can expose critical details, such as encryption keys or personal data, enabling attackers to breach system security. Common examples of SCAs include Timing Attacks^3^, Power Analysis Attacks^4^, Electromagnetic (EM) Attacks^5^, and Acoustic Attacks^6^, among others.

Side-channel attacks have increasingly targeted devices such as IoT^7^, gadgets, mobile devices, and embedded systems, as these often lack robust security measures to protect against these indirect threats. Mitigating side-channel attacks involves using countermeasures like data masking, noise injection, and dynamic reconfiguration, which disrupt the consistency of leaked information, making it harder for attackers to gain reliable data. Integrating side-channel resilience is crucial for securing cryptographic and critical systems against these covert cyber intrusions as technology advances.

FPGAs^8^ are highly configurable hardware devices, which means they can implement cryptographic algorithms and perform computations in parallel. While this flexibility is a significant advantage, it also introduces unique challenges for securing the system. Side-channel attacks represent a significant threat to FPGA design, particularly in applications like cryptography, where protecting sensitive data is crucial. FPGA has Dynamic Partial Reconfiguration (DPR)^9^, a unique capability that allows the same hardware to be used with different circuits at different times. DPR can be used to mitigate SCA on FPGA designs^10^. Also, FPGAs are very good at accelerating the Artificial Intelligence (AI) and Machine Learning (ML) models that enhance performance and energy efficiency^11^.

Research in FPGA design security^12^ is focused on defending against malicious attacks, as traditional security measures often struggle to keep pace with the evolving threat landscape. This highlights the need for innovative solutions to bolster the security of FPGA design systems. Deep Learning (DL) has emerged as a powerful approach in recent years, capable of learning complex patterns and representations from large datasets. DL has shown significant promise in enhancing the resilience of FPGA designs against various SCA^13^.

There was prior research to detect and mitigate the SCA on FPGA designs^14^. Still, they did not provide adaptive and comprehensive security solutions in real-time with the least feasible latency. To address the issues caused by SCA^2,8,9^ in FPGA designs, this work introduces a novel technique by combining the DL and DPR to detect and mitigate the SCA with the lowest latency and efficiency in real-time and it is scalable to address any kind of SCA. The contributions of this work are as follows:

1. Integrate a DL model and DPR to provide a comprehensive security solution against power SCA to demonstrate the concept

2. Detect and mitigate the SCA and measure the latency

The remainder of this document is as follows: Section II reviews relevant prior studies, Section III presents the proposed technique, Section IV details the findings, and Section V concludes with a summary and recommendations for future research.

## Literature review

This study provides an overview of existing literature on the evolution of SCA defense mechanisms in FPGA designs. It explores the most advanced methods currently employed to protect FPGA designs, aiming to identify gaps, challenges, and potential research directions for building secure and robust systems. Table 1 summarizes key studies on SCA and defense mechanisms in FPGA design security.

**Table 1:** An overview of the relevant research to mitigate SCA.

Author and Year	Methodology	Features	Challenges
P. Sasdrich et al. 2015^15^	PR	SCA resist Light Weight Cryptography (LWC) algorithm PRESENT	Not a comprehensive security solution against SCA. Limited to one LWC algorithm.
V. Bahadur et al. 2016^16^	PR	An implementation of SCA resistant True Random Number Generator (TRNG)	The application is limited to TRNG and does not offer a comprehensive security solution against SCA.
Pocklassery et al. 2017^17^	PR	An implementation of SCA resistant PUF	The application is limited to PUF and does not offer a comprehensive security solution against SCA. This is not an adaptive security solution.
P. Socha et al. 2019^18^	PR	Protect the cryptographic ciphers against the SCA	The application is limited to a few ciphers and does not offer a comprehensive security solution against SCA.
A. Kian et al. 2020^19^	DPR	DPA and CPA resist DPR based proposal	The application is limited to power SCA and does not offer a comprehensive security solution against SCA.
L. Bauer et al. 2024^20^	DL	Deep Learning-based SCA Detection for FPGA SoCs	This application is limited to detecting the SCA using DL methods and is not a comprehensive solution.
Monfared et al. 2024^21^	PR	Impedance SCA resist solution for the AES cipher	The application is limited to impedance SCA and does not offer a comprehensive security solution against SCA.
Proposed Work	DL & DPR	A heterogeneous approach using CNN-based DL to detect the SCA and DPR to mitigate it, keeping the system running seamlessly without compromising security.	A comprehensive and adaptive hardware security solution that mitigates SCA in real-time, and the proposed solution can be scalable to resist any kind of SCA in FPGA designs.

P. Sasdrich et al.^15^ leverage modern FPGAs’ Partial Reconfiguration (PR) capabilities to randomize cryptographic implementations’ hardware internals, thereby increasing resistance to SCA. The study focuses on the lightweight block cipher PRESENT as a case study. The authors utilize Configurable Look-Up Tables (CFGLUTs) available in modern Xilinx FPGAs to dynamically alter the configuration of the cipher’s hardware components during runtime. This dynamic reconfiguration makes it challenging for an attacker to predict the hardware state at any given time, mitigating the risk of SCAs. The implementation was tested on a Spartan-6 FPGA platform, and the results demonstrated that even after collecting 10 million power traces, no first-order leakage was detectable using state-of-the-art leakage assessment techniques. While the method was adequate for the PRESENT cipher, its applicability to more complex cryptographic algorithms remains to be thoroughly evaluated, and this is not a comprehensive solution.

V. Bahadur et al.^16^ propose a design that enhances resistance to side-channel attacks (SCAs) by leveraging reconfigurable hardware features. The proposed True Random Number Generator (TRNG) utilizes a Galois Ring Oscillator (GARO) structure, known for its robustness against SCAs. The design incorporates reconfigurable elements that adjust the oscillator’s configuration dynamically. This adaptability makes it more challenging for attackers to predict or manipulate the random number generation process. The implementation was tested on an FPGA platform, Altera Cyclone IV series and compliance with the NIST SP800-22 statistical test suite was demonstrated, indicating high-quality randomness. However, the proposal is limited to TRNG and lacks a comprehensive security solution.

Pocklassery et al.^17^ proposed the integration of Physical Unclonable Functions (PUF) and Dynamic Partial Reconfiguration (DPR) to enhance security in embedded systems. This proposal leverages PUFs for device authentication and key generation while using DPR to modify FPGA resources, dynamically making side-channel attacks more challenging. By combining these techniques, the study aims to improve the resilience of embedded systems against various security threats while maintaining efficiency and performance in constrained environments. However, the ability of the proposed security framework to adapt to new and evolving attack vectors remains uncertain, and continuous updates are necessary to maintain its effectiveness. It is not scalable or a comprehensive security solution to detect and mitigate all kinds of SCA.

P. Socha et al.^18^ proposed the application of DPR to enhance the security of two prominent block ciphers, AES and Serpent, against SCA. This approach involves modifying the hardware configuration during operation to introduce variability, making it more challenging for attackers to exploit side-channel information. The study demonstrates the effectiveness of this technique by implementing it on a Xilinx Spartan-6 FPGA. The results indicate that the fully protected versions of both AES and Serpent exhibit no significant leakage, suggesting robust resistance to SCA. The approach’s scalability to larger or more complex FPGA architectures has not been extensively evaluated and is not a comprehensive security solution.

Kian et al.^19^ propose utilizing FPGA devices’ DPR feature to obfuscate leaked information by dynamically altering the masking function during operation. This dynamic modification aims to reduce the effectiveness of SCA, particularly those targeting higher-order vulnerabilities. The methodology involves reconfiguring specific portions of the FPGA at runtime, thereby introducing variability that complicates the attacker’s ability to extract sensitive information. The effectiveness of this approach was evaluated through simulations and practical implementations, demonstrating its potential to enhance security in resource-constrained embedded systems. This proposal is not scalable for any kind of SCA and is not comprehensive. L. Bauer et al.^20^ proposed an innovative approach to enhancing the security of FPGA-based System-on-Chip (SoC) devices against SCAs. The authors propose a novel concept that utilizes DL techniques to detect power analysis (PA) and electromagnetic analysis (EMA) attacks. This approach aims to enable countermeasures only when necessary, thereby minimizing the associated power, performance, and area overheads. However, this methodology is not a comprehensive security solution for FPGA designs.

Monfared et al.^21^ present RandOhm, a defense mechanism that employs a moving target defense (MTD) strategy utilizing the partial reconfiguration (PR) capabilities of modern FPGAs and programmable SoCs. This approach involves dynamically reconfiguring the circuit’s placement and routing during operation, thereby randomizing the impedance characteristics of the power delivery network (PDN). By continuously altering the circuit’s configuration, RandOhm effectively decouples data-dependent computations from the PDN’s impedance, significantly reducing information leakage. The methodology was applied to AES cipher implementations on 28-nm FPGAs, demonstrating resilience against non-profiled and profiled impedance analysis attacks. Additionally, the study evaluates the performance overhead introduced by this mitigation strategy. This approach is not scalable to any kind of side-channel attack.

## Proposed methodology

This proposed work utilizes DL and DPR techniques that provide a comprehensive security solution to address the SCA in real-time.

**Fig. 1:**
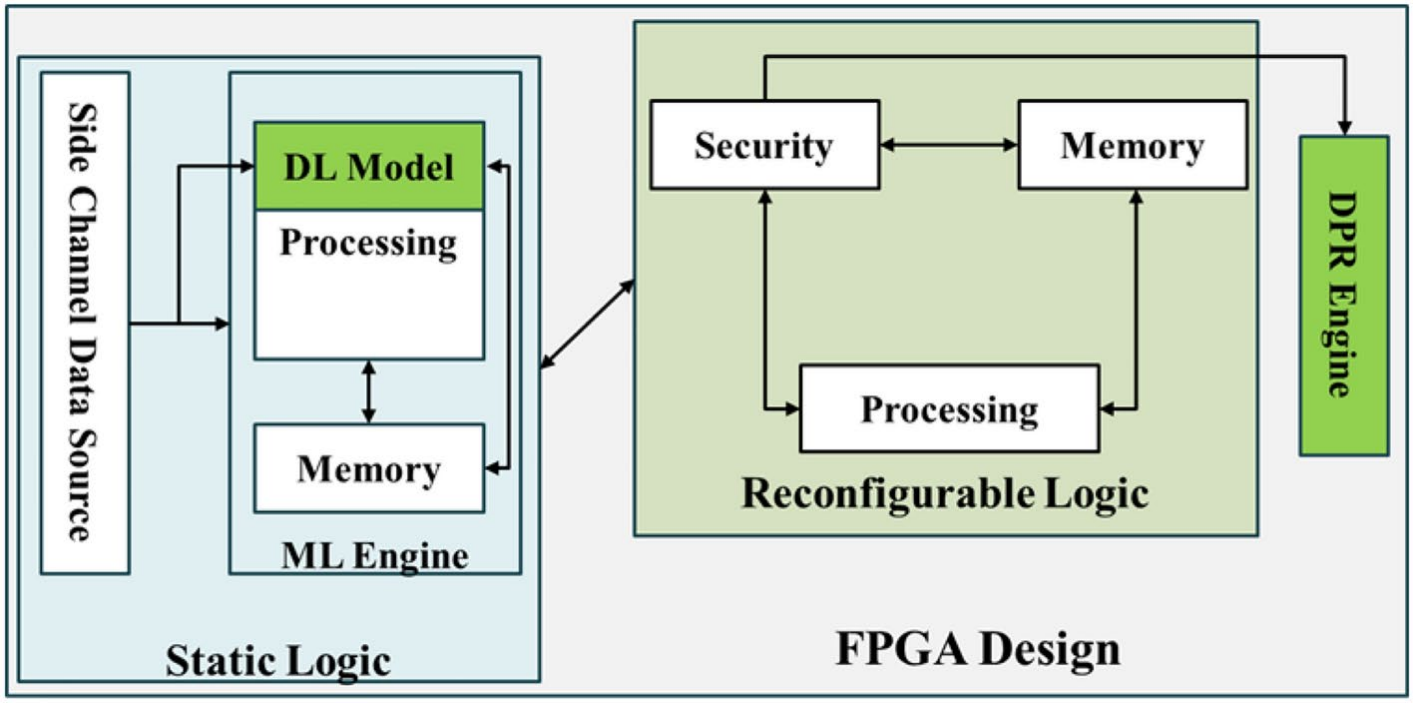
The architecture of Mitigating the SCA using the DL and DPR.

This proposed methodology is illustrated in Fig 1. The FPGA design has primarily two parts: A) Detect the SCA and B) Mitigate the SCA. The DL model in the “Static Logic” block detects the SCA and passes the information to the “Reconfigurable Logic,” where the cipher runs.

**Fig. 2:**
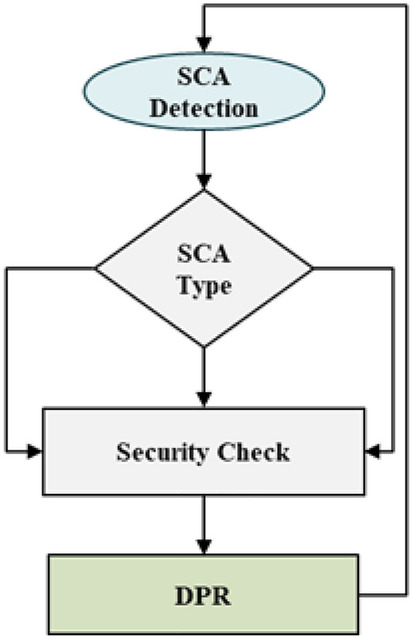
SCA detection and mitigation flow.

The DPR engine takes the request from the DL model and does the appropriate reconfiguration, which will change the “Reconfigurable Logic” hardware configuration without affecting its function. i.e., the reconfigured logic is functionally the same as earlier. Still, it differs in terms of number of gates, routing, consumption of power or placement location, and operating frequency, among others. The attacker aims to grab the security keys from the dynamic part of the FPGA design where the cipher and application run. The DPR reconfigures the hardware so that the attacker won’t be able to see helpful information that breaks the cipher. The flow of information is shown in Fig 2.

Fig 2 showcase the data flow end to end. The detection of the SCA then the information is analyzed to decide the kind of attack, and then it will go through a security check to determine whether the request came from the DL model. Then, it is passed to the DPR Engine to reconfigure the “Reconfigurable logic” based on the attack type.

Detecting a Side Channel Attack (SCA) using the DL model involves leveraging data from various sources that reflect unintended information leakage, which attackers exploit to infer sensitive information. The DL model continuously gathers side-channel information such as power consumption, electromagnetic emissions, execution time, or acoustic signals and extracts relevant features (e.g., statistical properties, signal patterns, frequency domain features) that differentiate normal and attack behaviors. After the feature extraction, the DL model was trained using the labeled dataset containing normal (benign) and attack (malicious) scenarios. The model is deployed to analyze the real-time data to detect the deviations or anomalies indicative of an SCA.

The model updates its training based on the learning from the inference in a feedback loop. Typically, side-channel data includes

1. Power traces: Variations in power consumption during cryptographic operations.
2. Electromagnetic emissions: Signals emitted due to electronic activity in the device.
3. Execution time: The time taken to execute specific operations may reveal sensitive details.
4. Acoustic emissions: Sounds produced during computation can carry data leakage.
5. Cache access patterns: Behavior of memory caches that may inadvertently expose data

Based on the above data, the DL model can detect Differential Power Attacks (DPA), Simple Power analysis (SPA), Timing Attacks, Cache Timing Attacks, and Electromagnetic Analysis (EMA), among others.

Dynamic Partial Reconfiguration (DPR) on FPGAs can mitigate SCA by altering the device’s functional layout during runtime. This approach dynamically reconfigures specific regions of the FPGA while the rest of the system continues to operate, reducing the predictability of operations exploited by SCA. DPR allows sensitive modules or cryptographic operations to be frequently reconfigured, modifying the physical implementation of the design. This disrupts the consistency of side-channel information, such as power consumption patterns or electromagnetic emissions, making it difficult for attackers to correlate data and extract sensitive information. Below are the advantages of DPR in mitigating the SCA.

1. Increased Unpredictability: Regular reconfiguration breaks the attacker’s models, increasing the effort and time required to exploit side channels.
2. Enhanced Security: Sensitive data and operations are better protected by continuously changing execution environments.
3. Efficient Resource Utilization: DPR enables time-multiplexing of FPGA resources, ensuring secure and efficient operation without requiring a larger FPGA.

The methodology outlined above combines the Convolutional Neural Network (CNN)^22^, a DL model, and DPR to prove real-time and intelligent hardware security against SCA. The reason for choosing CNN is its efficiency and strong performance on structured, sequential data:

1. Using convolutional filters, CNNs excel at capturing local patterns and features in time-series data, such as power traces^22^.

2. CNNs automatically extract hierarchical features, such as attack patterns, in side-channel data.

3. Many existing side-channel attack detection models are based on CNNs because power traces are often structured as sequential data

Integrating Dynamic Partial Reconfiguration and Deep Learning provides a dynamic, intelligent, and adaptive approach to countering SCA in FPGA designs. DPR ensures hardware-level obfuscation and adaptability, while DL models deliver robust detection and threat analysis. Together, they create a comprehensive framework for securing FPGA designs against side-channel threats. The combination of DPR and DL methods amplifies their effectiveness:

1. DL models monitor system behavior and detect SCAs in real-time.

2. Upon detection, DPR triggers reconfiguring the affected regions to neutralize the attack, randomize leakage patterns, or repair vulnerabilities.

3. This adaptive cycle strengthens security by proactively addressing threats and continuously evolving the system against sophisticated attacks, with better response time (latency).

## Results and discussions

**Fig. 3:**
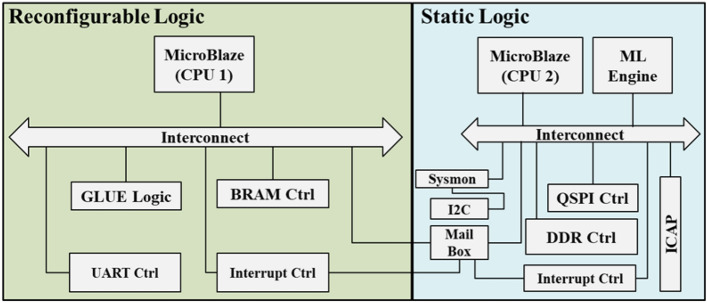
Proposed experimental design.

The proposed method was evaluated for detecting Simple Power Side Channel Attacks (SPA) using the VCU108 FPGA board^23^, as depicted in Fig 3. The FPGA design incorporates two CPUs: one implemented in Reconfigurable Logic (RL) and the other in Static Logic (SL).

The RL includes a BRAM controller for storing the AES application and keys and randomly configurable glue logic to vary the power consumption. Additionally, it features an interrupt controller to facilitate communication with the SL. The SL hosts the ML Engine, a hardware accelerator^24^, and executes the CNN model. It also integrates Sysmon for power monitoring and an I2C interface for communication with an external power regulator.

The DDR controller in the SL is the memory for the ML model parameters and the application that is responsible for Dynamic Partial Reconfiguration (DPR). The DPR process is executed through the Internal Configuration Access Port (ICAP)^25^. A mailbox mechanism and interrupts enable communication between the two CPUs (CPU1 and CPU2)^26^. The QSPI controller in the SL is responsible for storing the partial reconfigurable bitstreams.

The Clock Logic (CL) operates at a fixed frequency of 100 MHz, while the RL supports a configurable operating frequency ranging from 10 MHz to 100 MHz, adjustable in 5 MHz increments. Although the RL and CL operate at different frequencies, their clocks are synchronized.

**Fig. 4:**
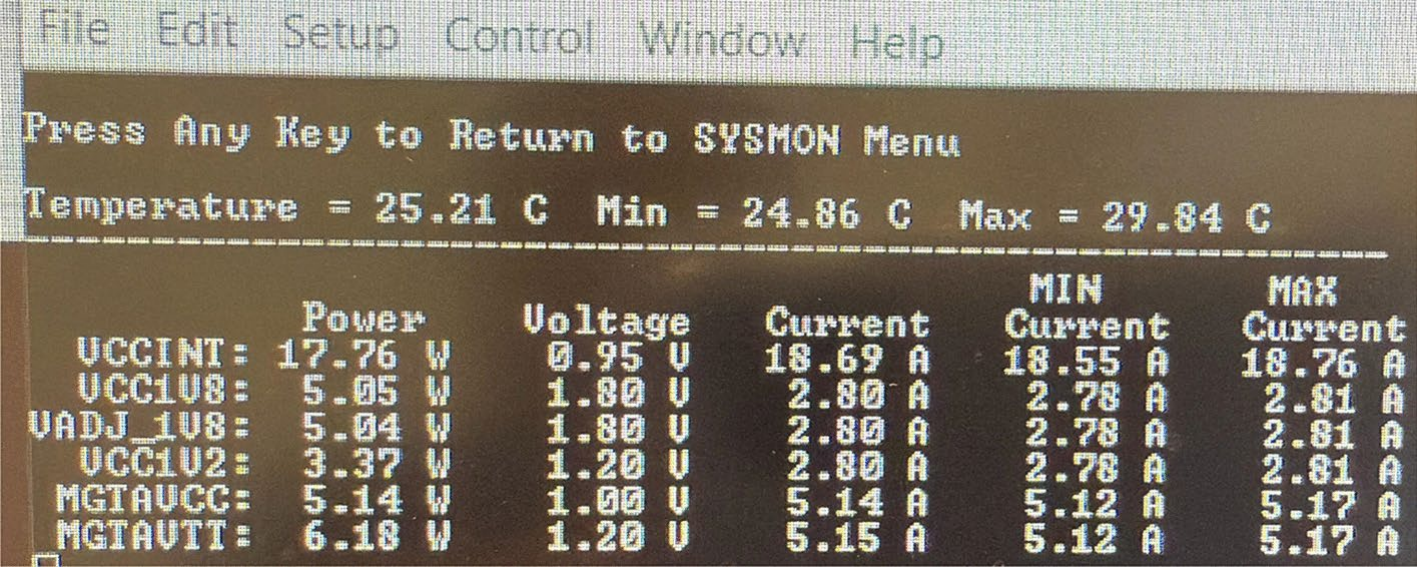
Continuous measurement of power.

The evaluation began with creating a comprehensive dataset by characterizing the behavior of a design implemented on the VCU108 board. This dataset was generated by leveraging the System Monitor (Sysmon) integrated into the VCU108 board, enabling real-time power consumption monitoring. Power consumption traces were recorded under regular operation (no attack) and attack conditions. These traces were captured using Sysmon and constituted a dataset representing normal and attack scenarios for the AES^27^ cipher. Various performance metrics^28^ were employed to evaluate the proposed CNN model’s effectiveness. These metrics provided a quantitative assessment of the model’s ability to accurately distinguish between attack and non-attack scenarios, demonstrating its potential to detect SPA threats. Fig 4 illustrates the variation in current while the AES cipher is running on CPU1. The continuous fluctuations in current during the cipher’s execution on the CPU were used to collect data for the attack and non-attack scenarios.

The performance of the proposed method was evaluated using the Receiver Operating Characteristic (ROC) curve and Area Under the Curve (AUC), a widely used metric to measure the performance of classification models. The model was trained using Jupyter Notebook. Additionally, the evaluation metrics outlined in^28^ were utilized to assess the model’s efficacy. These metrics provide a comprehensive view of the model’s ability to detect SCA:

1 $$\begin{aligned} & TPR = \frac{TP}{TP+FN} \end{aligned}$$

2 $$\begin{aligned} & \quad FNR = \frac{FN}{FN+TP} \end{aligned}$$

True Positive (TP), True Negative (TN), False Positive (FP), and False Negative (FN) are fundamental metrics for evaluating classification models. The True Positive Rate (TPR) measures the proportion of actual positives correctly identified by the model, reflecting the model’s ability to detect positive instances accurately. Conversely, the False Positive Rate (FPR) measures the proportion of actual negatives incorrectly classified as positives, indicating how often the model mislabels negative instances.

The relationship between TPR and FPR forms the basis of the Receiver Operating Characteristic (ROC) curve, a graphical representation of the trade-off between these metrics at various classification thresholds. The Area Under the Curve (AUC) quantifies the model’s overall performance, with higher AUC values indicating a better capacity to distinguish between classes.

**Fig. 5:**
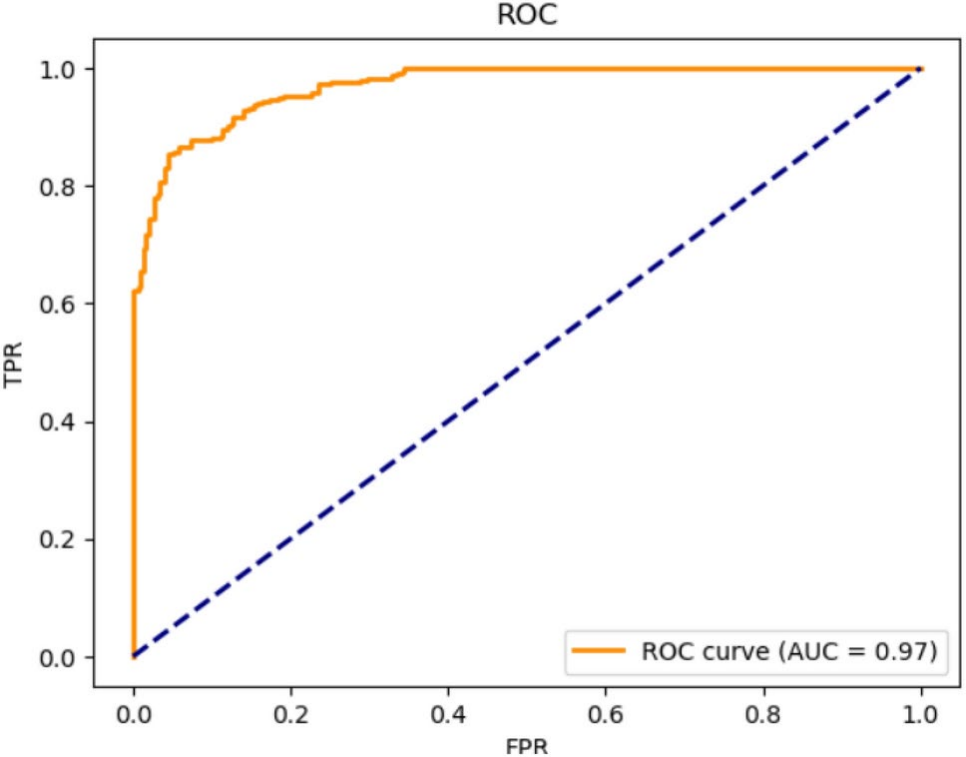
Receiver operating characteristic (ROC).

Fig 5 illustrates the ROC curve and AUC for the CNN model, demonstrating its effectiveness in differentiating between positive and negative instances under varying thresholds.

**Fig. 6:**
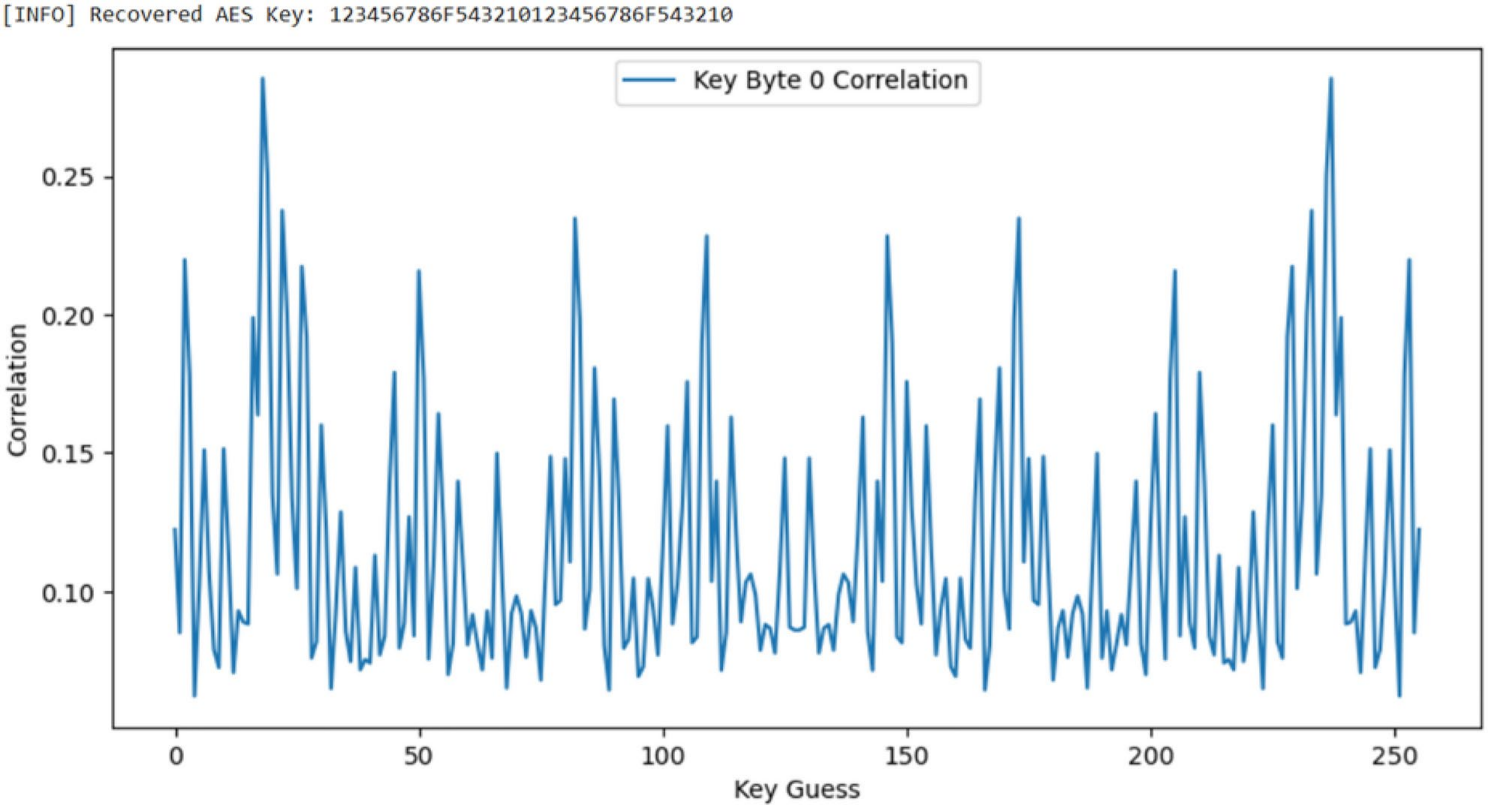
Pearson correlation analysis.

An additional check of the voltage drop across the shunt register on the line where the power was supplied from the regulator to the FPGA internal logic was captured, and the Pearson Correlation Analysis^29^ was performed to ensure the Side Channel Attack as shown in Fig 6.

The pre-trained model is integrated into the FPGA design, as shown in Fig 3. Vivado and Vitis AI tools were utilized to develop the FPGA design. The design is partitioned into two sections: Reconfigurable Logic (RL) and Static Logic (SL). The Details of the CNN layer are in Table 2. The CNN Model uses the Pooling and the dropout of 0.5.

**Table 2:** Layers of CNN model.

Layer	Type	Filters	Kernel Size	Activation
Conv1	Conv2D	32	(3,3)	Relu
Pool1	MaxPooling 2D	-	(2,2)	-
Conv2	Conv2D	64	(3,3)	Relu
Pool2	MaxPooling 2D	-	(2,2)	-
Conv3	Conv2D	128	(3,3)	Relu
Dense1	Dense	128	-	Relu
Dense2	Dense	10	-	Softmax

In the RL, the AES cipher is executed to encrypt and decrypt sample data that is read from the BRAM Controller. The DL model operates within the ML Engine and receives inference data from Sysmon, which provides power traces to the model while the AES cipher runs. The DL model analyses variations in the power traces and generates an alert to the CPU1.

Upon receiving the alert, the CPU1 acknowledges it and sends a “ready” signal to initiate DPR. The CPU2 triggers the DPR to reconfigure the RL based on the detected side-channel attack (SCA) type. The reconfiguration may involve various techniques [19] to mislead the attacker, depending upon the FPGA design’s ability to support it. However, this proposal uses the below methods for reconfiguration without affecting the overall functionality of the FPGA design as long as the design supports the changes.

### Clock gating

Disabling the clock to stop dynamic power, hindering attack analysis abruptly. The experiment was conducted without activating the DL model, and power traces were observed in an attempt to break the key used in the AES cipher. The experiment was then repeated with the DL model enabled, which successfully detected the side-channel attack (SCA) and initiated Dynamic Partial Reconfiguration (DPR). The DPR was configured for clock gating dynamically. As a result of this reconfiguration, the attacker could not obtain meaningful information as the clock was dynamically gated and ungated. The observed power consumption was limited to the static leakage power of the logic.

### Dynamic frequency adjustment

Stepping up or down the clock frequency to alter power consumption patterns. The experiment was then repeated with the DL model enabled, which successfully detected the side-channel attack (SCA) and initiated Dynamic Partial Reconfiguration (DPR). The DPR was applied by dynamically stepping up/down the clock frequency from 10 MHz to 100 MHz. The resulting power consumption values are varied, and the attacker could not extract any information to reveal the key.

### Dynamic logic addition

Dynamic Partial Reconfiguration (DPR) is employed as the glue logic to modulate the power consumption of the FPGA design. The glue logic implemented within the FPGA consists of random sequential logic blocks that are strategically designed to consume varying amounts of power. These logic elements are interconnected via a bus interface, enabling seamless interaction with the interconnect fabric of the FPGA. The design of the glue logic incorporates multiple levels of combinational and sequential logic, which can be selectively activated or deactivated. This flexibility allows for controlled power consumption variations without interfering with the primary functionality of the FPGA, particularly the execution of cryptographic operations. The core functionality of this approach lies in periodically reconfiguring the glue logic using DPR every 1 millisecond (ms). The reconfiguration process replaces existing logic with new randomly generated logic structures, altering the overall power consumption of the FPGA fabric at regular intervals. This periodic adjustment results in a continuous variation of power levels, making it difficult to establish a stable power signature that attackers could exploit. As the AES algorithm executes on CPU1, the DPR-based glue logic simultaneously undergoes frequent reconfiguration. This causes substantial fluctuations in power consumption, masking the actual power usage patterns of the cryptographic computations. Since side-channel attacks, such as power analysis attacks, rely on correlating power traces with computational operations, the introduced power variations significantly disrupt these correlations. Consequently, adversaries attempting to analyze power consumption patterns to extract meaningful information, such as intermediate values used in encryption, are unable to derive accurate data for cryptographic key recovery.

The results demonstrate that integrating DL models with DPR on FPGAs offers an innovative and practical approach to counter SCAs. While the DL models provide real-time detection and adaptive responses, DPR introduces hardware variability and on-demand deployment of countermeasures. This combination not only enhances the security of FPGA designs but also ensures the efficient use of resources, offering a dynamic and scalable solution to mitigate side-channel vulnerabilities, making it a promising approach for securing cryptographic systems in the evolving threat landscape. An advantage of this approach is that DPR was selectively activated only when a side-channel attack was detected, unlike prior research, as discussed in 1. Experimental results indicate that the latency from detecting the SCA to activating the mitigation is within 20 clock cycles, which is not feasible in prior research discussed in 1.

### Comparison to the state of art

In conclusion, we assess our proposed framework against state-of-the-art research, focusing on FPGA resource utilization and performance as shown in Table 3. While our approach is not a direct comparison with prior works, the primary objective of this study is to showcase the seamless integration of Side-Channel Attack (SCA) detection using an AI model with Dynamic Partial Reconfiguration (DPR) for real-time mitigation.

A key factor in securing embedded systems against SCAs is latency, as mitigation must be activated almost immediately upon detection. Our results demonstrate that the time from detection to the activation of mitigation is achieved within just 20 clock cycles, ensuring rapid and efficient response to potential threats.

**Table 3:** Comparison with the state of the art.

Approach	ML based [20]	Proposed method
Calculation time	45$\upmu$s	10 ms
Detection metric	Supply voltage variation	Supply voltage variation
Technology	16nm	20nm
Area (kGE)	107.47 + ARM CPU based PS [30]	3000
Accuracy	0.90	0.97
Latency (clocks)	-	20
Evaluation	Hardware tested	Hardware tested

Additionally, our framework is designed to be highly resource-efficient, enabling the entire AI model to be deployed on a low-end FPGA while the supporting software runs on a MicroBlaze soft processor. This stands in contrast to prior work, such as [20], where the implementation was carried out on a ZCU104 FPGA^30^ with an ARM Cortex processor running at 1.2 GHz, which is part of the ASIC section of the FPGA. By utilizing a low-power and cost-effective FPGA solution, we demonstrate that high-performance SCA detection and mitigation can be achieved even in resource-constrained environments, making our approach suitable for a wider range of applications.

## Conclusion

This paper presents a novel adaptive security framework that integrates Deep Learning (DL) with Dynamic Partial Reconfiguration (DPR) to detect and mitigate Side-Channel Attacks (SCAs) on FPGA-based systems. By leveraging the strengths of AI-driven detection and real-time hardware reconfiguration, the proposed approach ensures a robust, efficient, and low-latency security mechanism. The AI model serves as the core detection engine, continuously analyzing side-channel data for patterns and anomalies that may indicate potential attacks. This enables real-time, intelligent threat detection with minimal computational overhead. Once an SCA is identified, DPR dynamically reconfigures critical regions of the FPGA, disrupting predictable leakage patterns that attackers exploit. This adaptive defense mechanism not only mitigates the risk of SCAs but also enhances the overall resilience of FPGA-based designs. By combining AI-powered detection with hardware-level reconfiguration, the proposed methodology achieves a highly adaptive security framework that ensures minimal latency from attack detection to mitigation. Our results demonstrate that mitigation can be triggered within just 20 clock cycles, significantly reducing the window of vulnerability while maintaining optimal resource utilization. Furthermore, the proposed solution is scalable and generalizable, making it applicable to various SCA scenarios and security-sensitive applications. Unlike prior research that relies on high-end ARM processors on advanced FPGAs (e.g., ZCU104 with an ARM CPU at 1.2 GHz), our implementation demonstrates that effective SCA detection and mitigation can be achieved on a low-end FPGA with a MicroBlaze soft processor, thereby reducing hardware costs and power consumption. This work does not seek to identify the best ML model or DPR methodology but rather to demonstrate the feasibility and effectiveness of integrating these techniques into a unified, real-time security framework. By proving that AI-driven detection and DPR-based mitigation can seamlessly coexist, this study lays the foundation for future advancements in adaptive security mechanisms for FPGA-based systems. Our approach paves the way for the development of scalable, intelligent, and resource-efficient security solutions capable of countering evolving SCA threats in diverse domains.

## Data Availability

All results and design details are fully presented and described within the paper.
